# Structural, Optical, and Antibacterial Efficacy of Pure and Zinc-Doped Copper Oxide Against Pathogenic Bacteria

**DOI:** 10.3390/nano11020451

**Published:** 2021-02-10

**Authors:** Awais Khalid, Pervaiz Ahmad, Abdulrahman I. Alharthi, Saleh Muhammad, Mayeen Uddin Khandaker, Mubasher Rehman, Mohammad Rashed Iqbal Faruque, Israf Ud Din, Mshari A. Alotaibi, Khalid Alzimami, David A. Bradley

**Affiliations:** 1 Department of Physics, Hazara University, Mansehra 21300, Pakistan; awais.phy@hu.edu.pk (A.K.); saleh@hu.edu.pk (S.M.); 2 Department of Physics, University of Azad Jammu and Kashmir, Muzaffarabad 13100, Pakistan; 3 Department of Chemistry, College of Science and Humanities, Prince Sattam Bin Abdulaziz University, P.O. Box 173, Al-Kharj 11942, Saudi Arabia; a.alharthi@psau.edu.sa (A.I.A.); i.din@psau.edu.sa (I.U.D.); alosaimi@psau.edu.sa (M.A.A.); 4 Center for Applied Physics and Radiation Technologies, School of Engineering and Technology, Sunway University, Bandar Sunway 47500, Selangor, Malaysia; mayeenk@sunway.edu.my (M.U.K.); d.a.bradley@surrey.ac.uk (D.A.B.); 5 Department of Microbiology, Hazara University, Mansehra 21300, Pakistan; mubasherrehman08@gmail.com; 6 Space Science Centre, Universiti Kebangsaan Malaysia (UKM), Bangi 43600, Selangor, Malaysia; rashed@ukm.edu.my; 7 Department of Radiological Sciences, College of Applied Medical Sciences, King Saud University, PO Box 10219, Riyadh 11433, Saudi Arabia; kalzimami@ksu.edu.sa; 8 Department of Physics, University of Surrey, Guildford GU2 7XH, UK

**Keywords:** copper oxide, zinc-doping, hydrothermal, antibacterial activity

## Abstract

Copper oxide and Zinc (Zn)-doped Copper oxide nanostructures (CuO-NSs) are successfully synthesized by using a hydrothermal technique. The as-obtained pure and Zn-doped CuO-NSs were tested to study the effect of doping in CuO on structural, optical, and antibacterial properties. The band gap of the nanostructures is calculated by using the Tauc plot. Our results have shown that the band gap of CuO reduces with the addition of Zinc. Optimization of processing conditions and concentration of precursors leads to the formation of pine needles and sea urchin-like nanostructures. The antibacterial properties of obtained Zn-doped CuO-NSs are observed against Gram-negative *(Pseudomonas aeruginosa, Klebsiella pneumonia, Escherichia coli)* and Gram-positive *(Staphylococcus aureus)* bacteria via the agar well diffusion method. Zn doped s are found to have more effective bacterial resistance than pure CuO. The improved antibacterial activity is attributed to the reactive oxygen species (ROS) generation.

## 1. Introduction

The manipulation of size, composition, and morphology of technologically significant materials ranging from nanometer to micrometer has been a major challenge for scientists over the past several decades [1]. Metal oxide nanostructures possess special physical and chemical properties due to their finite size and large surface area. Metal oxides have attracted great interest from scientists due to their multi-discipline applications [2,3,4]. Copper oxide (Cu) is among the most significant p-type semiconductors, having Cu atoms on Face centered cubic (FCC) and oxygen (O) atoms at the center with a band gap of 1.2 eV–1.9 eV [5,6]. CuO-based nanomaterials are extensively studied and used in various applications due to their unique properties, i.e., high physical and chemical stability, non-toxicity, narrow optical band gap, infrared photodetection, and ferromagnetic behavior (because of its opposite surface spin) [7,8,9,10]. Moreover, in certain semiconductors and oxide-based materials, optical, magnetic, and electronic properties are often observed [7]. The three most important oxidation states of copper is Cu^+^, Cu^2+^, and Cu^3+^, causing the possibility to dope electrons and holes [8]. These properties make CuO an ideal contender for a variety of applications, including gas sensors, solar cell, supercapacitors, lithium-ion electrode resources, superconductors, magnetic storage media, and catalysis. So far, various copper oxide nanostructures (CuO-NSs) have been reported, including one dimensional, two dimensional, spherical, and hierarchical structures [11,12,13,14]. Cu is a well-known antibacterial agent due to its high efficacy against bacteria [15,16]. CuO nanoparticles kill bacteria by the release of Cu^2+^ ions [17]. ZnO is also known for its significant inhibition of bacterial growth in a wide range of bacteria, caused by the production of reactive oxygen species (ROS) formed in water [18,19]. During interaction with water, ZnO reduces oxygen to water and produces three intermediate ROS; hydroxyl radical, hydrogen peroxide, and superoxide [20,21]. These types of species play a key role in killing gram-positive and gram-negative bacteria. The ROS generation in a nanomaterial structure by zinc oxides depends on the existence of defect sites [22].

Zn^2+^ dopant has been documented to be the most successful in producing defects in CuO nanostructures that might have the potential for biological applications [23]. Due to Cu^2+^ (0.072 nm) and comparable ionic states being almost the same ionic radius of Zn (0.074 nm), Zn^2+^ is promising in various fields. Zn-doped CuO oxide has been reported as an excellent candidate for anti-bacterial resistance. Zn-CuO nanoparticles with antibacterial drugs could be deposited on the cotton substrate to attain an anti-bacterial cotton bandage [24]. The combined antibacterial properties of CuO and ZnO can be investigated by doping Cu into the matrix of ZnO or doping Zn into the matrix of CuO. The production of strong resistance pathways to such hybrid metal particles becomes a challenge for bacteria [25].

Various preparation methods for nanostructures are described in the literature, such as sol-gel combustion, co-precipitation, chemical vapor deposition, laser ablation, hydrothermal treatment, ball mill, and microwave-assisted synthesis, etc. [26,27]. An economical and environmentally-friendly method was developed for the synthesis and functionalization of copper oxide (CuO) nanosheets by chemical grafting of 3-(chloropropyl) triethoxysilane (ClPTES), diethanolamine (DEA), and *p*-amino thiophenol (ATP), with potential uses in catalysis and biomedical applications [28]. Silver-doped CuO was found to have promising prospects for potential applications as an inexpensive catalyst in wastewater treatment and antibacterial agent in cosmetics [29]. The antibacterial activity of Mg-doped ZnO nanoparticles (NPs), synthesized by the green chemistry route using malabathricum leaf extract [30], and size-dependent activity of ZnO-NPs, synthesized by various concentrations of Mar Ivanios leaf extract, were tested against different clinical strains. It was observed that as the concentration of NPs increases, the antibacterial activity also increases [31]. The ZnO-CuO composites synthesized using colotropis gigantea leaf extract by a combustion method shows good antibacterial activity [32]. The synthesis of a Zinc (Zn)-doped Copper oxide nanostructure (Zn-doped CuO-NS) and its chemical and physical properties, including its crystal structure and magnetization, has been explored as a function of temperature [33]. Similarly, it was found that doping of magnesium in CuO-NSs possesses anti-cancer and antimicrobial activity for various microbial strains [34]. There have also been reports on the antimicrobial activity of Zn-doped CuO-coated fabrics experienced against *E. coli*, *S. aureus*, and multidrug-resistant bacteria [24].

Previously, many researchers have carried out doping with transition metal in CuO using various methods, but only a few of these works are about Zn-doped CuO-NSs. The structural, morphological, optical, and antibacterial properties of nanomaterials depend mainly on the impurities and defects existing in a host matrix. The modification in the properties of CuO is observed by introducing dopants into the lattice. Thus, this study examined the effect of pure and Zn-doped CuO-NSs on gram-positive bacterium (*S. aureus*) and gram-negative bacterium (*P. aeruginosa, K. pneumonia, E. coli*). We observed enhanced killing of gram-negative and gram-positive bacterial strains by the Zn-doped CuO-NS.

## 2. Materials and Methods

### 2.1. Materials

Zinc chloride (ZnCl_2_), Copper II chloride dihydrate (CuCl_2_∙2H_2_O), and Potassium hydroxide (KOH) were purchased from Sigma Aldrich, St. Louis, MI, USA. Structural analysis of Zn-doped CuO samples was carried out by using an X-ray diffractometer (Ultima IV Rigaku International Corp., Tokyo, Japan), scanned and recorded at 20–70° at CuK radiation (λ = 1.54056 Å). The surface morphology of pure and Zn-doped CuO-NSs was examined with a FE-SEM (QUANTA 250 FEI). X-ray photoelectron spectroscopy (XPS; Thermo specific model K-ALPHA) was used to investigate the surface elemental composition. To perform the antibacterial activity, four different bacterial strains were used, namely, *Pseudomonas aeruginosa* (*ATCC^®^ 10145*)*, Klebsiella pneumonia* (*ATCC^®^ BAA-1144*)*,* and *Escherichia coli* (*ATCC^®^ 33876*) as gram-negative and while *Staphylococcus aureus* (*ATCC^®^ 11632*) as gram-positive bacteria. Nutrient agar (Oxoid^®^ CM0003) was purchased from Sigma-Aldrich.

### 2.2. Synthesis of Zn-Doped CuO-NSs

CuCl_2_. 2H_2_O, ZnCl_2_, and KOH were taken as precursors. At first, a mixture of 3.4 g of CuCl_2_·2H_2_O was dissolved into 40 mL of deionized water. For the doped sample, 0.85 g of ZnCl_2_ was added along with CuCl_2_·2H_2_O. Afterward, 2.7 g of KOH was separately dissolved in 40 mL of deionized water and added dropwise to the already made homogeneous solution of CuCl_2_·2H_2_O. At room temperature, the solution was stirred for 30 min. The final solution was transferred to a (Teflon-lined) autoclave then placed in the oven at 180 °C for 18 h. As a result, a dark brown and aquamarine precipitate was obtained for pure and Zn-doped samples. The as-obtained dark brown and aquamarine precipitate was then washed many times with distilled water and ethanol. Finally, the precipitate was kept for drying in the oven at 120 °C for 2 h.

### 2.3. Screening of Antibacterial Activity

For screening of antibacterial efficacy of CuO and Zn-doped CuO-NSs, all bacterial strains were sub-cultured from their pure cultures in Luria broth media (containing 17% glycerol) and subjected to overnight incubation. For the antibacterial assay, the fresh cultures were used by transferring the stock suspensions on nutrient agar (Oxoid^®^ CM0003) and incubated at 37 °C for 24 h. Bacterial culture turbidity was settled to the 0.5 McFarland (freshly prepared) standard [35], which is equal to 1.5 × 10^8^ CFU/mL bacteria. Every species was spread on a nutrient agar Petri plate with the help of a sterile glass spreader. By using a sterile polystyrene tip, 4 mm wells were rendered. Pure and Zn-doped CuO-NSs with various concentrations, including 3, 5, and 10 mg/mL, were prepared in 20% dimethyl sulfoxide (DMSO). In each well, 40 µL concentration was added, which was taken from the prepared solution. All the plates were placed in an incubator overnight at 37 °C for incubation. By using a caliper, the inhibition zone was measured in millimeters around each well. Ciprofloxacin, as a standard reference antibiotic, was used at a 40 µg/mL concentration. The mean value was reported for each experiment, carried out in triplicate (N = 3).

## 3. Results and Discussion

**Figure 1:**
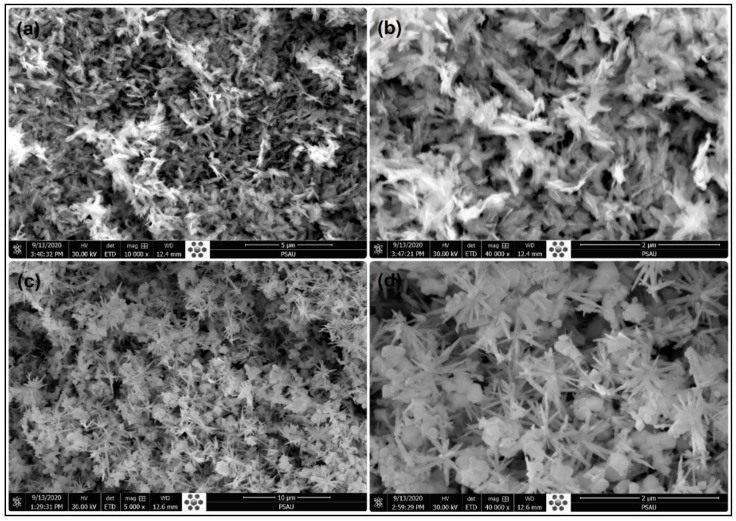
FE-SEM micrographs of the (**a**,**b**) pure copper oxide and (**c**,**d**) shows the micrographs of Zinc (Zn)-doped Copper oxide (Zn-doped CuO).

The characterization of hydrothermally prepared CuO and Zn-doped CuO-NSs was performed by using a field emission scanning electron microscope (FE-SEM) to observe its apparent shape. Figure 1a,b shows the FE-SEM micrographs of the synthesized pure CuO in low and high magnifications. Similarly, Figure 1c,d shows the FE-SEM micrographs of Zn-doped CuO-NSs in lower and high magnifications. The morphologies of both pure and Zn-doped CuO-NSs were very interesting. The structure of pure CuO seemed to be feather-like and changed to a pine needle-, sea-urchin- [36], or block-like structure with Zinc doping. The change in the morphology might be due to Cu^2+^ (0.73 Å) replacement with Zn^2+^ (0.74 Å) in the CuO lattice. Feather-like structures in pure CuO had a thickness in the range of 50–100 nm. The pine needle-like structures in the Zn-doped sample varied in the range of 80–100 nm, whereas the block-like structures were found to vary in size from 80–200 nm. The observation of the FE-SEM results shows the variation in size and morphology of the structures with doping of Zn. The size and shape of CuO-NSs were found to depend on the Zn additive. These findings were found to be in close agreement with the previous findings [37].

**Figure 2:**
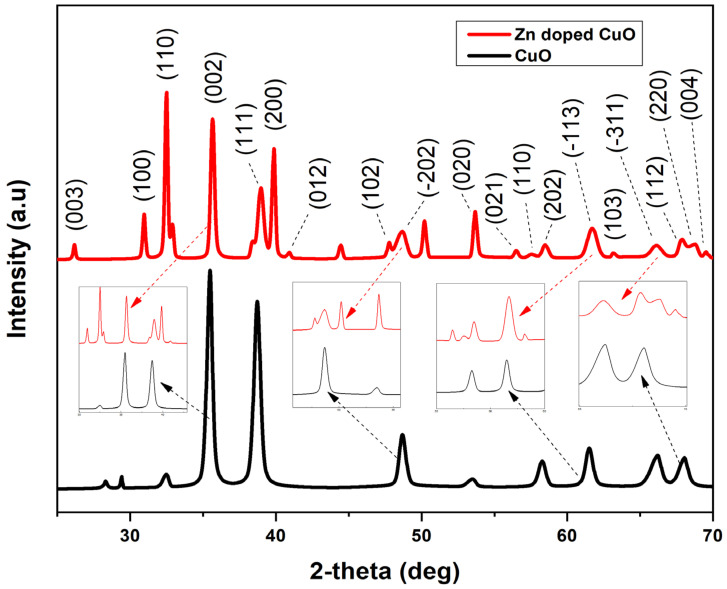
XRD pattern of Zn-doped CuO nanoparticles showing peaks for different content in the sample.

The x-ray diffraction (XRD) spectrum of the synthesized CuO and Zn-doped CuO-NSs were displayed in Figure 2. The compositions, phase, and crystallite size of the material were determined by intensities and peak positions of the observed peaks. The peaks in the displayed XRD pattern appeared at 2θ different values. According to “Inorganic crystal structure database (ICSD) reference No. 01-080-1268”, the peaks found at 29.42°, 32.53°, 35.5°, 38.7°, 48.6°, 53.51°, 58.29°, 61.51°, 66.17°, and 68.07° corresponded to (210), (110), (002), (111), (−202), (020), (202), (−113), (−311), and (220) planes in the monoclinic structure of CuO [38,39,40]. The sharpness of the peaks indicated that pure CuO was highly crystalline. In Zn-doped CuO, the XRD pattern peaks appeared at a 2θ value of 26.20°, 30.97°, 32.42°, 35.64°, 38.97°, 39.86°, 40.97°, 47.74°, 48.59°, 53.6°, 56.41°, 57.52°, 58.41°, 61.74°, 63.68°, 66.17°, 67.85°, 68.73°, and 69.40°, respectively. These peaks, according to “ICSD reference No. 001-1136”, corresponded to (003), (100), (110), (002), (111), (200), (012), (102), (−202), (020), (021), (110), (202), (−113), (−311), (112), (220), and (004) planes [41], which shows that the Zn^2+^ ions were incorporated effectively to the site of CuO lattice without interrupting the CuO crystal structure. Due to the resemblance in the Zn^2+^ (0.74 Å) and Cu^2+^ (0.73 Å) radius, Cu^2+^ ions can be well substituted by Zn^2+^ ions in the lattice structure.

**Figure 3:**
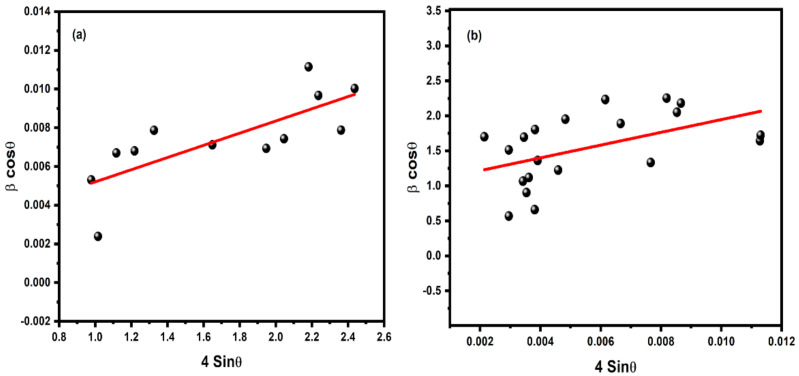
Williamson-Hall (W-H) analysis for (**a**) CuO and (**b**) Zn-doped CuO nanostructures.

**Figure 4:**
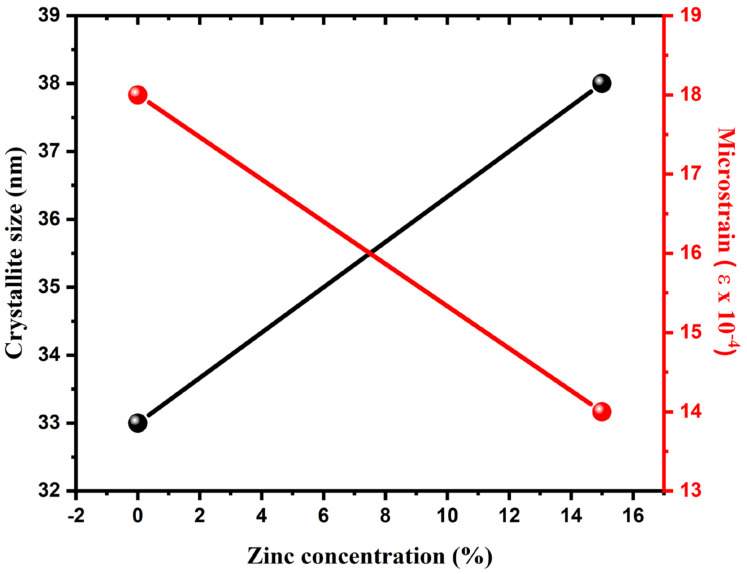
Analysis of microstructural parameters, such as crystallite size D and micro-strain (ε), with respect to Zinc concentration for the synthesized samples.

The particle size and strain were investigated by using the Williamson-Hall (W-H) method, as displayed in Figure 3, for pure and Zn-doped CuO-NSs. The W-H method varies with tanθ instead of 1/cosθ, as followed in the Debye Scherrer equation. This essential variation allows one to separate the broadening of reflection, along with both microstructural reasons (micro-strain and small crystallite size) that occur together. The microstructural parameters were calculated, including the size of crystallite D and micro-strain (ε) for the prepared samples. It was found that the average size of CuO crystallites increased and ε decreased with increasing Zn concentration, as shown in Figure 4. Compared to the core ions, this property could be due to the slightly greater ionic radius of the dopant.

**Figure 5:**
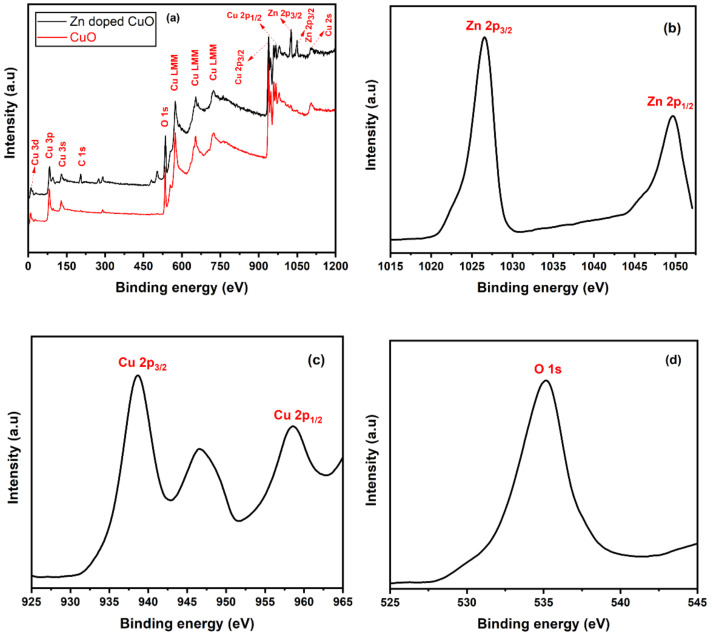
(**a**) X-ray photoelectron spectroscopy (XPS) survey of the as-synthesized Zn-doped CuO nanostructures. (**b**) High resolution Zn 2p, (**c**) Cu 2p, and (**d**) O 1s XPS spectra.

X-ray photoelectron spectroscopy (XPS) was carried out to confirm the composition and valence states of the synthesized nanostructures, as shown in Figure 5. The survey of prepared Zn-doped CuO-NSs in Figure 5a shows Auger peaks for Zn 2p, Cu 2p, C 1s, and O 1s. The presence of the C1s peak in the survey spectrum was due to surface contamination. The peaks that appeared in Figure 5b were assigned to Zn 2p_3/2_ and Zn 2p_1/2_, which were positioned at 1024.3 eV and 1047.7 eV. This indicates that Zn oxidized (Zn^2+^) in the CuO nanostructures and was substituted at the Cu^2+^ site into the CuO lattice. The binding energy of major peaks, such as Cu 2p_3/2_ and Cu 2p_1/2_, appeared at 937.5 eV and 958.4 eV, with a spin-orbit splitting of about 20.1 eV, as shown in Figure 5c. Moreover, the presence of two satellite peaks indicated the existence of Cu^2+^ located at about 10 eV higher than those of the Cu 2p_3/2_ and Cu 2p_1/2_ [42,43]. The magnified O 1s peak is shown in Figure 5d. The broad peak was assigned to O^2−^ ions at a binding energy of 533.2 eV in the Cu–O bonding of the monoclinic structure in oxygen-deficient regions [44]. XPS results confirm the structure consisting of a CuO-NS doped with Zn was in good agreement with the literature data [45,46].

**Figure 6:**
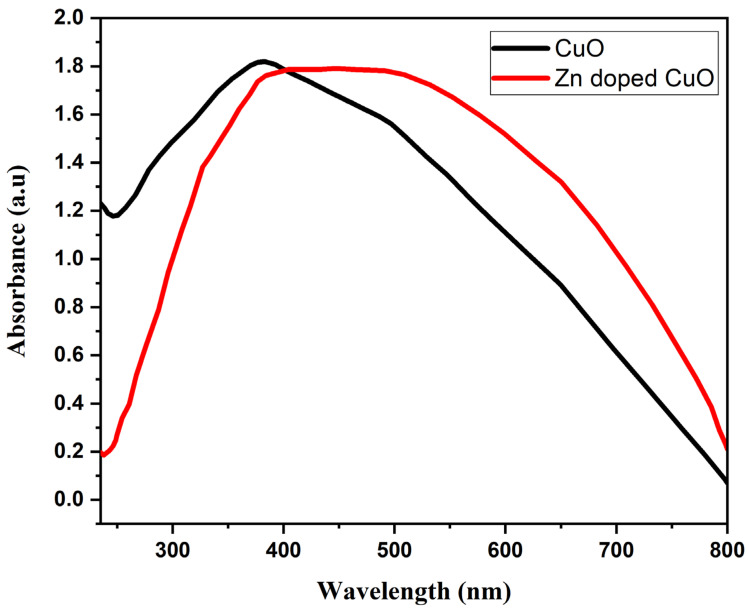
UV-visible absorption spectra of pure and Zinc (Zn)-doped copper oxide nanostructures (Zn-doped CuO-NSs).

**Figure 7:**
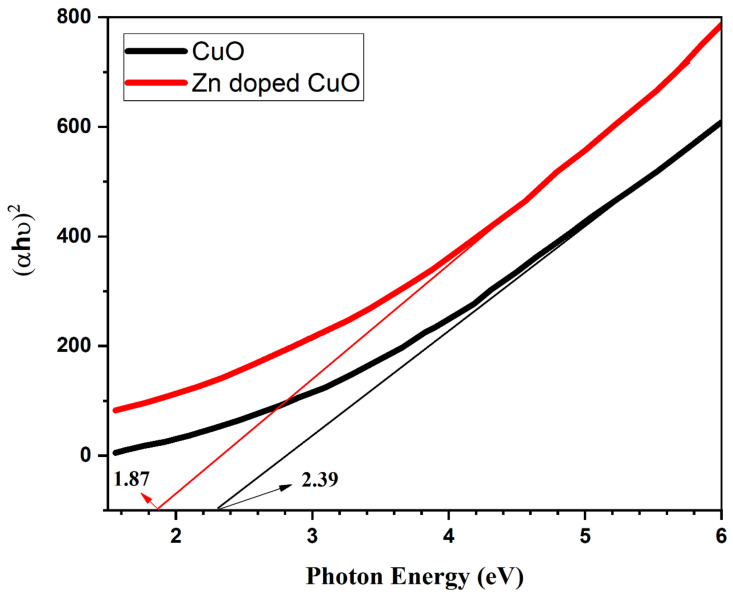
Band gap absorption edges of CuO and Zn-doped CuO nanostructures.

The absorption spectrum of the CuO and Zn-doped CuO-NS is shown in Figure 6. The absorption properties for the band gap of nanostructures were recorded in the 230–800 nm range. The absorption peak for the CuO-NS was observed at 390 nm, which moves toward the visible region with Zn doping. The visible light absorption capability was enhanced with Zn doping, which can be a potential for photocatalysis. The d-d transition among closely spaced Cu^2+^ and Zn^2+^ ions was responsible for the enhancement in the absorption of light in the visible region with Zn doping [47]. The optical band gap (Eg) of the Zn doped CuO-NS was measured using the Tauc relation [48]. The band gap of obtained samples was measured by plotting (αhν)^2^ versus the energy of incident photons (i.e., E = hν), as shown in Figure 7. There was an Eg of 2.39 eV in the CuO-NS, which indicates important quantum confinement effects in comparison with bulk CuO (1.55 eV) Eg [49]. This reduction in Eg can be correlated with the effect of quantum confinement [49]. The same Eg value was recorded earlier for the CuO-NS [50]. Zn doping into a CuO-NS resulted in a decrease of Eg to 1.82 eV for the Zn-doped CuO-NS [51]. The reduction of Eg can also be considered as a result of the transition from oxygen 2p state to d state of Cu and Zn. Finally, we came to the conclusion that high concentration CuO-NSs doped with Zn had a good optical property comparable to pure CuO-NSs [47].

The antibacterial potential of synthesized CuO-NSs doped with Zn was observed by the agar well diffusion method [52] against American type culture collection (ATCC) bacterial strains collected from the Department of Microbiology, Hazara University Mansehra. A total of 4 bacterial strains (*Pseudomonas aeruginosa* (*ATCC^®^ 10145*), *Klebsiella pneumonia* (*ATCC^®^ BAA-1144*), and *Escherichia coli* (*ATCC^®^ 33876*) as gram-negative bacteria and *Staphylococcus aureus* (*ATCC^®^ 11632*) as gram-positive bacteria) were used for this study. Through various biochemical tests, all bacterial strains were identified, according to the method described [53]. A pure culture of bacteria was stored in agar slants at 4 °C for later use.

**Figure 8:**
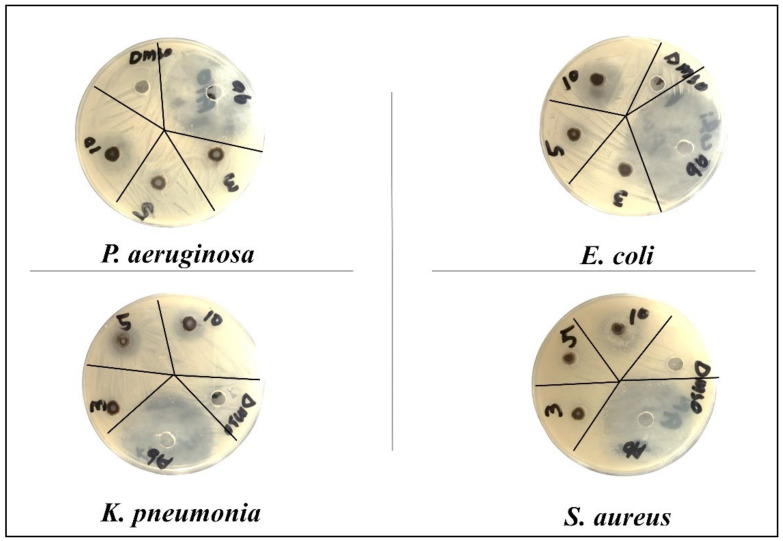
Zone of inhibition formed by CuO-NSs against different bacteria.

**Figure 9:**
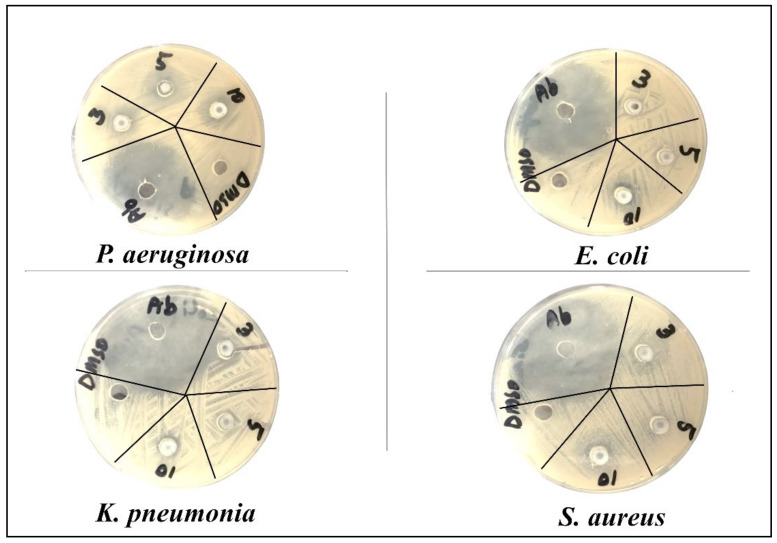
Zone of inhibition formed by Zn-doped CuO-NSs against different bacteria.

**Figure 10:**
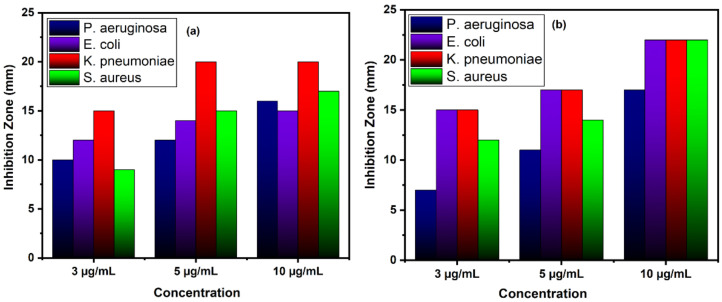
Bar graph showing the diameter of the zone of inhibition (in mm) produced by (**a**) pure and (**b**) Zn-doped CuO nanoparticles (NPs) against gram-positive and gram-negative bac-tria.

The cultures were treated with various doses of pure and Zn-doped CuO-NSs (3, 5, 10 mg/mL) dissolved in 20% DMSO. The results demonstrate that Zn-doped CuO-NSs inhibited the growth of all the tested microbes in all the tested doses, as shown in Figure 8 and Figure 9. The zone of inhibition (ZOI) increases with an increase in the concentration of Cu-doped ZnO-NSs. We observed that gram-positive microbes were more susceptible to pure and Zn-doped CuO-NSs as compared to gram-negative microbes. Among gram-negative microbes, *P. aeruginosa* formed a ZOI of 16 ± 0.21 mm and 17 ± 0.14 m and, *E. coli* formed 15 ± 0.20 mm and 22 ± 0.21 mm ZOI for CuO and Zn-doped CuO, respectively. *K. pneumonia* was more sensitive to Zn-doped CuO-NS treatment and displayed 20 ± 0.20 mm and 22 ± 0.20 mm ZOI. Gram-positive microbes, such as *S. aureus*, formed a ZOI of 17 ± 0.13 mm and 22 ± 0.13 mm on the same dose for pure and Zn-doped CuO-NSs, as shown in Table 1. All the results were carried out in triplicate, and the mean diameter of the inhibition zone was recorded and evaluated by using SPSS version 25 as shown in Figure 10a,b. Inhibition zone vs concentration bar graphs shows the diameter of the inhibition zone produced by CuO and Zn-doped CuO-NSs against gram-positive and gram-negative bacterial strains. The enhanced antibacterial activity as a result of Zn doping in CuO-NSs is clearly observed against all microbial strains.

## 4. Conclusions

Pine-needle- and sea urchin-like pure and Zn-doped CuO-NSs were prepared by a simple hydrothermal route. A comparative study of the nanostructures of pure and Zn doped CuO was carried out to observe the stimulating effect of Zn doping on optical and antibacterial characteristics. According to x-ray diffraction analysis, Zn-doped CuO-NSs had some additional peaks compared to CuO, which indicates that the Zn^2+^ effectively replaced Cu^2+^ in CuO crystal lattice. CuO and Zn-doped CuO-NSs were found to inhibit the growth of all tested bacteria. The inhibitory effect is enhanced in a dose-dependent manner. It has been observed that gram-positive microbes are more susceptible to Zn-doped CuO-NSs than gram-negative microbes. It is observed that Zn dopant brings great improvement in the antibacterial activity of CuO-NSs. This study indicates that doping is a successful strategy for the growth of the most efficient antimicrobial material.

## Figures and Tables

**Table 1:** The table summarizes the detail of bacteria and other experimental parameters.

Bacteria			CuO			Zn Doped CuO		
			3 mg/mL	5 mg/mL	10 mg/mL	3 mg/mL	5 mg/mL	10 mg/mL
Gram negative	*P. aeruginosa*	Inhibition zone (mm)	10 ± 0.12	12 ± 0.20	16 ± 0.21	7 ± 0.15	11 ± 0.13	17 ± 0.14
	*E. coli*		12 ± 0.11	14 ± 0.11	15 ± 0.20	15 ± 0.21	17 ± 0.11	22 ± 0.21
	*K. pneumoniae*		15 ± 0.20	20 ± 0.15	20 ± 0.20	15 ± 0.12	17 ± 0.20	22 ± 0.20
Gram positive	*S. aureus*		9 ± 0.13	15 ± 0.11	17 ± 0.13	12 ± 0.14	14 ± 0.18	22 ± 0.13

## Data Availability

All the data is available within the manuscript.
